# Metal-organic frameworks for hepatocellular carcinoma therapy and mechanism

**DOI:** 10.3389/fphar.2022.1025780

**Published:** 2022-09-26

**Authors:** Yingqi Feng, Wei Wu, Muzi Li

**Affiliations:** Beijing Key Laboratory for Green Catalysis and Separation, Department of Environmental Chemical Engineering, Beijing University of Technology, Beijing, China

**Keywords:** hepatocellular carcinoma, MOFs, photothermal therapy, photodynamic therapy, chemodynamic therapy

## Abstract

In recent years, metal organic frameworks (MOFs) have attracted increasing attention in cancer therapy, because they can enhance the anticancer efficacy of photodynamic therapy (PDT), photothermal therapy (PTT), photoacoustic imaging, and drug delivery. Owing to stable chemical adjustability, MOFs can be used as carriers to provide excellent loading sites and protection for small-molecule drugs. In addition, MOFs can be used to combine with a variety of therapeutic drugs, including chemotherapeutics drugs, photosensitizers, and radiosensitizers, to efficiently deliver drugs to tumor tissue and achieve desired treatment. There is hardly any review regarding the application of MOFs in hepatocellular carcinoma. In this review, the design, structure, and potential applications of MOFs as nanoparticulate systems in the treatment of hepatocellular carcinoma are presented.

**Systematic Review Registration**: website, identifier registration number

## Graphical Abstract

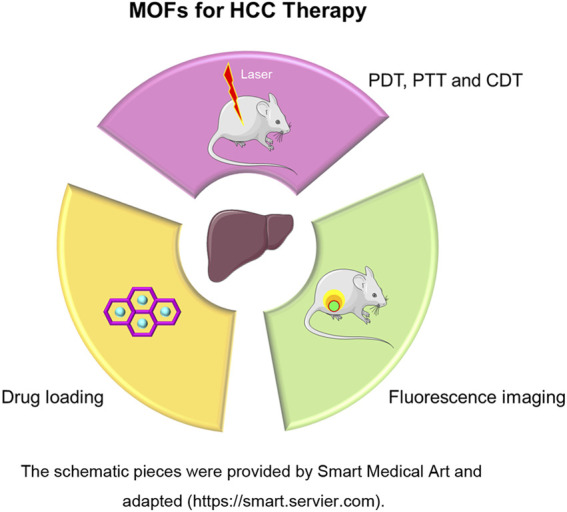

## Introduction

Hepatocellular carcinoma (HCC), a disease with a high incidence rate, will lead to 41,260 new diagnoses and 30,520 new deaths in the United States in 2022, according to the predictions of the National Cancer Institute. Accounting for 5% of all cancer deaths, the 5-year survival period for HCC is only 20.8%. Currently, surgery, radiotherapy, and chemotherapy based on targeted drugs are used to treat HCC (Chen et al., 2020; Rinaldi et al., 2021). Many patients with HCC do not have obvious symptoms, are usually diagnosed at an advanced stage, and are not suitable for surgery or transplantation (Du et al., 2021). Under the circumstances, chemotherapy, radiation therapy, and other treatment methods can effectively prolong the survival of patients and improve their quality of life (Yang et al., 2020; Zheng et al., 2021). However, chemotherapy or radiotherapy treatment faces many challenges, such as individual differences in HCC patients, poor sensitivity to chemotherapy or radiation therapy, and drug resistance (Mao et al., 2022). Furthermore, large doses of chemotherapy or radiotherapy can produce serious side effects, resulting in poor compliance of patients (Feng Y et al., 2020). Therefore, looking for new therapeutic methods with new mechanism, reducing the dose of drugs, and maintaining antitumor efficacy are urgent problems that must be solved in the treatment of HCC (Hao Y.N et al., 2021; Philips et al., 2021).

Metal organic frameworks (MOFs) are a new form of coordination polymer developed in recent years, which are self-assembled involving metal ions and organic molecules. They have advantages such as porous structure, good biocompatibility, large specific surface area, and easy modification. They are widely used in the fields of catalysis (Sadeghi and Sillanpää, 2021; Zhang et al., 2021), energy storage (Downes and Marinescu, 2017; Jayaramulu et al., 2021), separation (Li T et al., 2022), and biomedicine (Carrillo-Carrión, 2020; Chen J et al., 2021). In cancer treatment, MOFs can enhance the anticancer effects of PDT, PTT, chemodynamic therapy (CDT), photoacoustic imaging, and drug delivery (Chen J et al., 2021). Additionally, MOFs have good chemical adjustability and can be used as carriers to provide excellent loading sites and protection for small-molecule drugs. MOFs can be combined with a variety of therapeutic drugs, including chemotherapeutics, photosensitizers (Luo et al., 2021; Zhao et al., 2021), and radiosensitizers, to efficiently deliver drugs to tumor sites (Osterrieth and Fairen-Jimenez, 2021) and achieve effective treatment (Carrillo-Carrión, 2020).

PDT is a novel tumor intervention approach that can replace traditional antitumor methods (Lakshmi and Kim, 2019). The mechanism of PDT produced by MOFs is to provide MOF molecules (photosensitizers) with specific structures such as porphyrins in tumor tissue and then locally irradiate the tumor with light of specific wavelength to excite the photosensitizer (Neufeld et al., 2021). The excited photosensitizer will transfer its energy and electron to the surrounding oxygen atoms, thereby generating singlet oxygen and other reactive oxygen species (ROS), and then kill tumor cells (Feng G et al., 2020; Chen D et al., 2021). Unlike chemotherapeutics and radiotherapy, which can cause systemic toxicity, oxygen substances produced during PDT treatment have no toxic effect on the body. Due to a series of advantages, such as noninvasive, small side effects, and accurate administration, PDT has been widely used in the treatment of superficial tumors and adjuvant treatment after surgical resection of tumors (Yu et al., 2020).

PTT, characterized by low systemic toxicity and efficient targeted local treatment, can ablate tumor cells with heat generated by a specific MOF after near-infrared (NIR) radiation. This approach is an extension of photodynamic therapy, in which a photosensitiser is excited with specific band light. This activation brings the sensitiser to an excited state where it then releases vibrational energy (heat) and kills the targeted cells (Xiong et al., 2021; Dai et al., 2022). Furthermore, the combination of PDT and PTT can synergistically improve antitumor efficacy and reduce side effects. The heat generated by PTT can improve blood flow and oxygen supply, thus improving the sensitivity of tumor cells to oxygen-dependent PDT. Furthermore, ROS produced by PDT can interfere with tumor physiology and change the microenvironment, thus improving the thermal sensitivity of tumor cells (Sun X et al., 2021; Zeng et al., 2021).

CDT is an effective strategy to inhibit tumor cells by converting H_2_O_2_ into highly toxic •OH through Fenton or Fenton-like reaction (Liu et al., 2021a). In the Fenton reaction generated by MOFs, Fe^2+^ in MOFs acts as a catalyst to convert H_2_O_2_ into highly toxic •OH. In addition to Fe^2+^, generated Cu^2+^, Mn^2+^, and CO^2+^ can also catalyze the formation of •OH through Fenton-like reactions. In view of the characteristics of •OH produced during CDT, its combination with PDT can enhance the efficacy of PDT through the O_2_ produced, and the production of highly toxic •OH can also kill cells with O_2_, further inhibiting tumor cells. In recent years, PDT/CDT combination therapy has been methodically explored to enhance tumor oxidative stress and obtain better antitumor effect compared to monotherapy (Hao X et al., 2021; Tian et al., 2021).

Due to the advantages of the MOFs structure, several studies have attempted to utilize MOFs for anticancer applications. Such MOFs often are exploited for phototherapy, imaging effects, and drug loading functions. These studies have not only developed a variety of antitumor drugs with different structures but have also made remarkable achievements in drug delivery, loading and targeting (Lawson et al., 2021; Ma et al., 2021).

## MOFs-mediated PDT, PTT and CDT treatment of HCC

**FIGURE 1:**
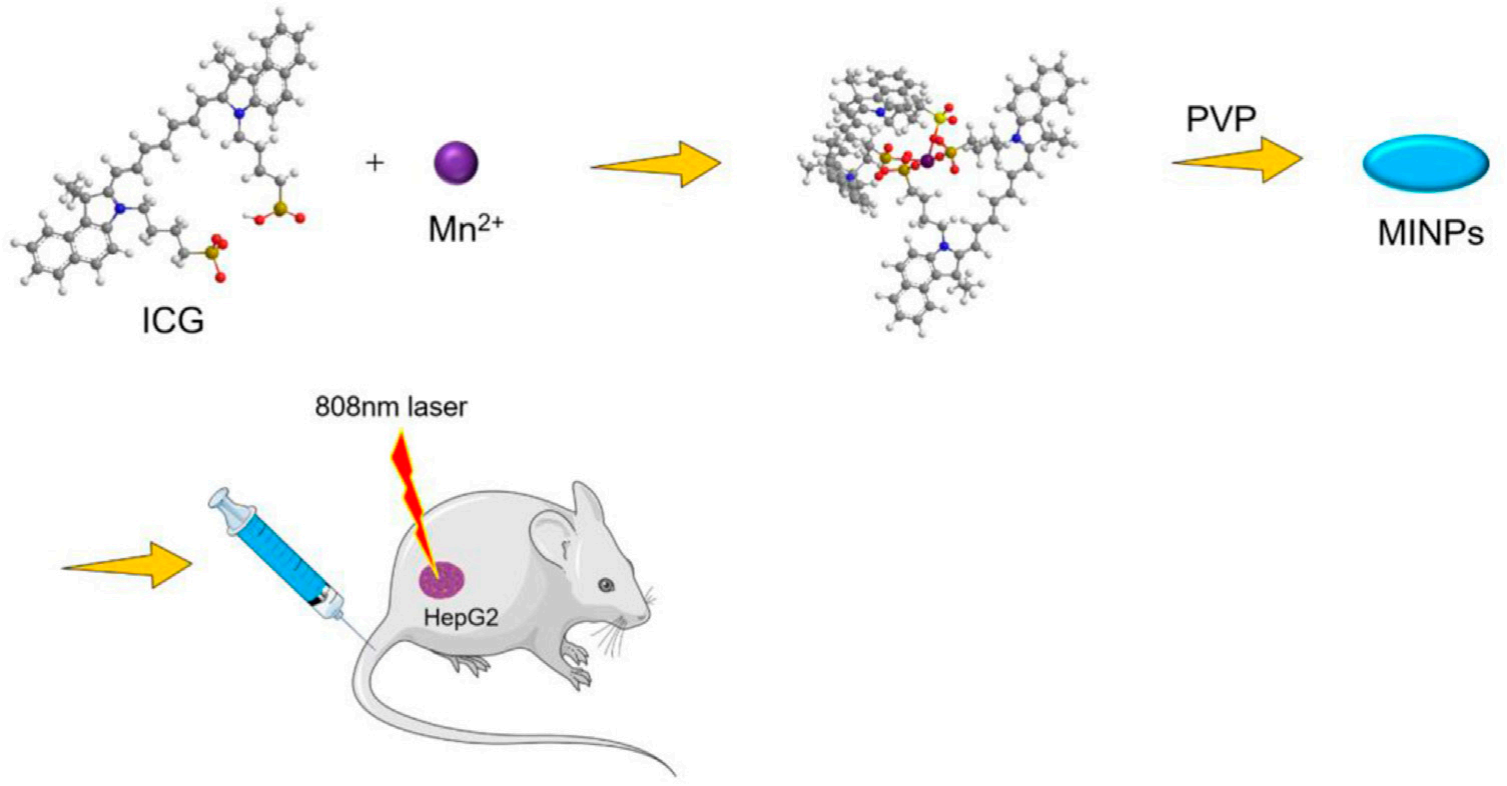
Synthesis and application of MINP. The schematic pieces were provided by Smart Medical Art and adapted [http://www.servier.com]. Servier Medical Art by Servier is licensed under a Creative Commons Attribution 3.0 Unported License.

Some MOFs with special structures can kill tumor cells through PDT, PTT or CDT effect. Therefore, MOFs have been applied in tumors treatment (Sun X et al., 2021; Wang et al., 2021; Zhou et al., 2021). Shi et al. (Shi et al., 2018) used a simple method to assemble Mn^2+^ and ICG and constructed new self-assembled nanoparticles (MINPs) under the protection of polyvinylpyrrolidone (PVP). *in vitro* experiments, under laser irradiation at 808 nm, MINPs achieved a clear inhibitory effect on HepG2 cancer cells (Figure 1). The MINPs solution was injected into the subcutaneous tumor model of mice, the photoacoustic (PA) signal around the tumor tissue during a 12-h period was recorded. The intensity of the PA signal obtained in the injection group was about three times higher than that of the control group (ICG administration group). After injection of MINPs, the positive signal of tumor magnetic resonance imaging (MRI) was enhanced and the average signal intensity gradually increased, indicating that MINPs achieved a time-dependent tumor accumulation. The quantitative detection results showed that the intensity of the MRI signal after 12 h of injection was 1.8 times higher than before injection. The results of *in vitro* PTT treatment and *in vivo* imaging revealed that cell necrosis and tumor damage could be observed in the tumor (HepG2 cells) treated by MINPs, while the control group did not present any obvious tumor damage. These experiments indicated that MINPs could be expected to become a highly effective PTT therapeutic drug and could be applied for imaging-guided PTT of HCC.

**FIGURE 2:**
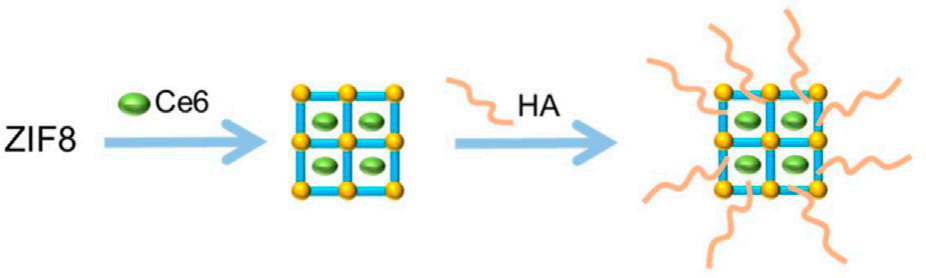
Synthesis of ZIF-8@Ce6–HA.

Fu et al. (2020) developed a new therapeutic agent ZIF-8@Ce6-HA using a one-step method. In this therapeutic agent, ZIF-8 was loaded with Chlorin E6 (CE6) and then modified with hyaluronic acid (HA) ZIF-8@Ce6-HA exhibited a good encapsulation rate, cell absorption, and biocompatibility. Mass spectrometry test data showed that HA modification prolonged the blood circulation time of these particles and decreased toxicity (Figure 2). *In vitro* anticancer experiments showed that after 5 min of irradiation at 660 nm, free CE6 exhibited slight cytotoxicity at a higher concentration (3 mM), and approximately 29.5% of HepG2 cells died due to ROS generated by PDT. Free CE6 molecules tend to agglomerate in the aqueous phase to reduce the efficiency of PDT, while ZIF-8@Ce6-HA overcomes this problem. The ZIF-8@Ce6-HA group showed greater cytotoxicity than the free CE6 group after irradiation with death of all cancer cells (88.4%). That study innovatively introduces a new therapeutic agent CE6 for PDT.

Liu et al. (2017) constructed MOFs with biocompatible Zr ions and Meso-Tetra (4-carboxyphenyl) porphine (TCPP), then loaded doxorubicin (DOX) to build a new nanoparticle (NP), DOX@NPMOF. The content of DOX loaded on the particles was as high as 109%. In an *in vitro* HepG2 cell model, DOX@NPMOF administration with a 655 nm laser showed good inhibitory ability (IC_50_ = 67.72 μg/ml) and the lethality of HepG2 cells was as high as 90%. Subsequently, a mouse subcutaneous tumor model was used to investigate the anticancer effect of DOX@NPMOF. The experiment was divided into four groups, namely, the saline group, the PDT treatment group, the chemotherapy group, and the combined treatment group (DOX@NPMOF), to compare the results. First, the fluorescence intensity of the cancer area was recorded by imaging. After the accumulation of NPMOF reached its maximum, the tumor tissues of mice in each group were irradiated with a 655 nm laser (180 J/cm^2^) for 15 min. Subsequently, the size of the tumor in different groups was monitored. The experimental results showed that the combined treatment group achieved the best effect. After 2 days of treatment, the tumors of mice were significantly reduced and gradually completely eradicated. The effects obtained were superior to that of the chemotherapy group and the PDT treatment group, and no skin/tissue damage was observed in any mice.

**FIGURE 3:**
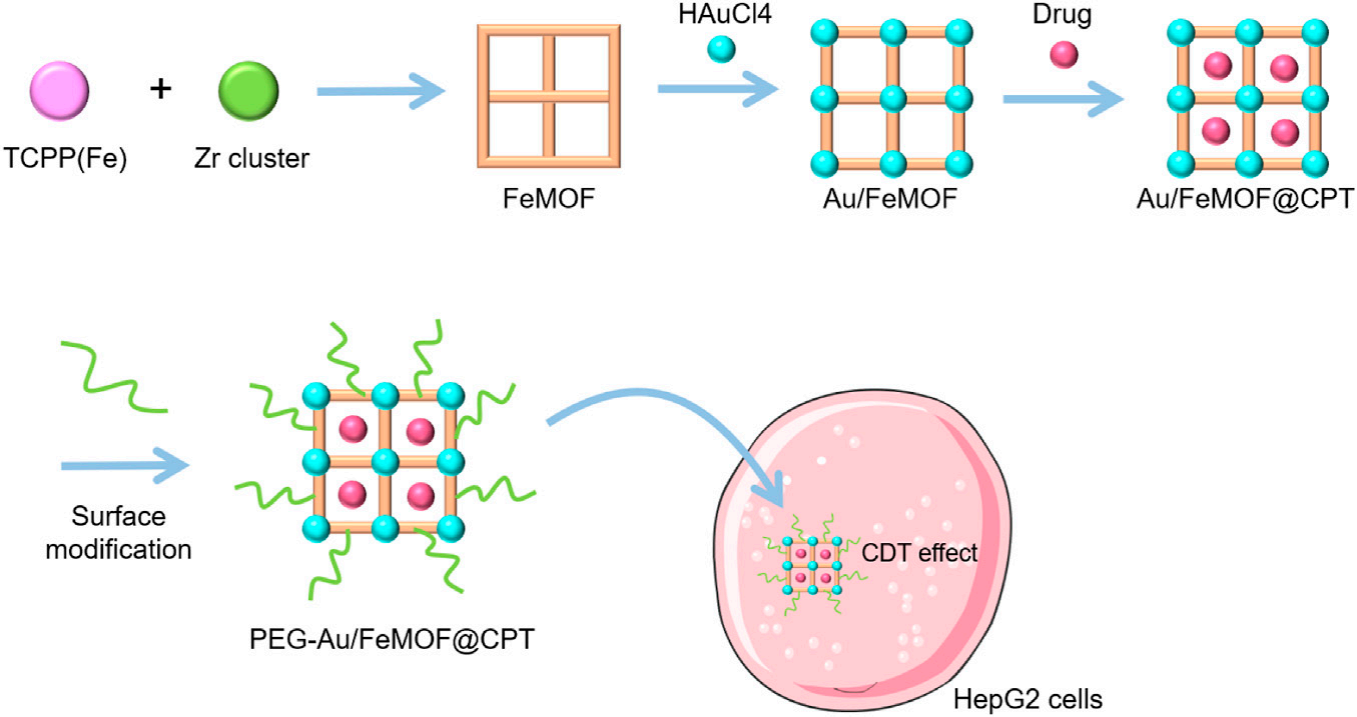
Synthesis and application of PEG-Au/FeMOF@CPT NPs. The schematic pieces were provided by Smart Medical Art and adapted [http://www.servier.com]. Servier Medical Art by Servier is licensed under a Creative Commons Attribution 3.0 Unported License.

Ding et al. (2020) constructed FeMOFs nanoparticles (NPs) with TCPP (Fe) and zirconium clusters, then loaded the hydrophobic chemotherapeutic drug camptothecin (CPT), and *in situ,* grew small gold (Au) NPs on its surface to gain novel NPs PEG-Au/FeMOF@CPT NPs. The Au NPs externally anchored were further modified by 1-dodecyl mercaptan (C_12_SH) and methoxypolyethylene glycol mercaptan (PEG-SH) (Figure 3). The MTT assay was used to evaluate the anticancer effects of CDT on HepG2 cells. The half maximum inhibitory concentration (IC_50_) of CPT monotherapy was 206 ± 22 μg/ml. Although the amount encapsulated in CPT was only 7.7%, the IC_50_ value of the chemokinetic treatment group (PEG-Au/FeMOF-NPs) still reached 3.51 ± 0.26 μg/ml. Conversely, the IC_50_ value of the combined chemotherapy and chemokinetic treatment group (PEG-Au/FeMOF@CPT NPs) was the lowest (0.31 ± 0.04 μg/ml). This indicates that the combination of CDT and CPT may achieve a good synergistic activity and can effectively inhibit tumor growth.

## MOFs for synergistic cancer treatment

Chemotherapy, one of the main methods for treating tumors, uses chemical synthetic drugs to kill cancer cells. Some chemotherapeutic drugs, such as DOX and CPT can be combined with MOFs to overcome the problems of poor drug release, side effects of systemic administration, and drug resistance, to enhance the antitumor effect. MOFs achieve high drug loading by changing the binding sites and porosity, promoting accumulation in the tumor, and prolonging the drug release by appropriate modification. Therefore, MOFs achieve good synergistic effects with chemotherapy drugs to achieve antitumor goals.

**FIGURE 4:**
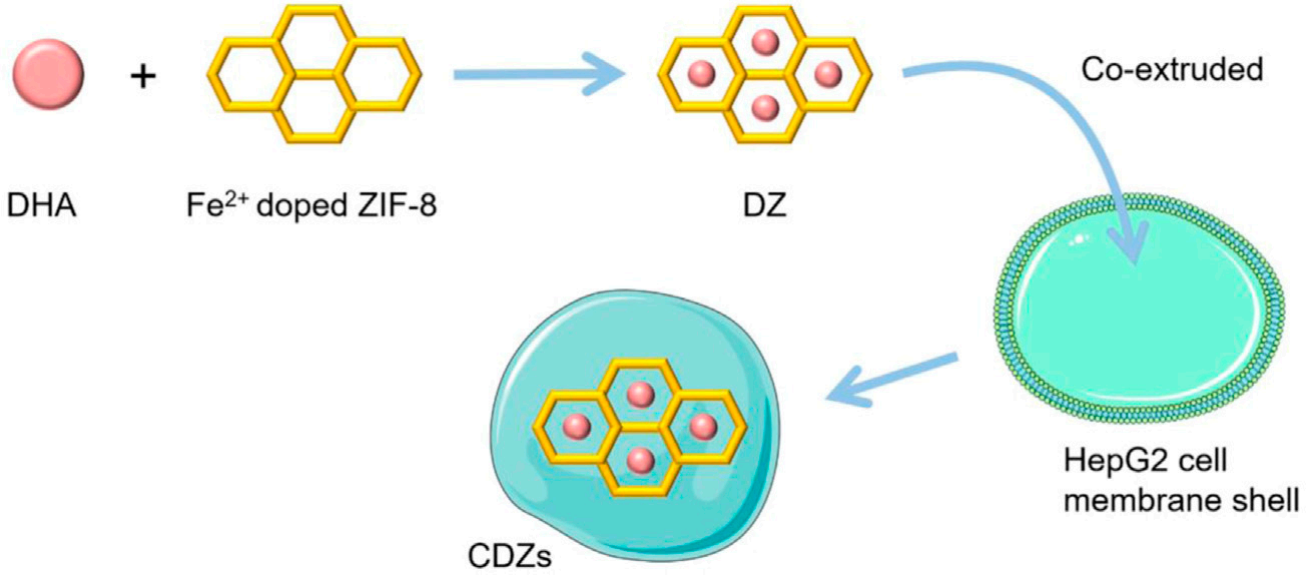
Synthesis and application of CDZs. The schematic pieces were provided by Smart Medical Art and adapted [http://www.servier.com]. Servier Medical Art by Servier is licensed under a Creative Commons Attribution 3.0 Unported License.

Xiao et al. (2020) designed a biomimetic MOF particle (CDZ) loaded with dihydroartemisinin (DHA) based on the ZIF-8 structure. They first prepared Fe^2+^ doped ZIF-8 NPs using a simple method and loaded DHA into the NPs to form DZs. The DZs were then inserted into the shell prepared with cancer cell membrane to obtain CDZs, which able to accumulate and be released into the tumor tissue. When Fe^2+^ in particles combine with DHA, the hydrogen peroxide bridge will fracture reductively and oxygen center free radicals will be generated, which will lead to rearrangement of carbon center free radicals and induce DHA toxicity. DHA influences the mitochondrial-dependent apoptosis pathway and can inhibit the activation of the nuclear factor kB (NF-kB) signaling pathway, thus promoting tumor cell apoptosis (Figure 4).

*In vivo* antitumor effects of CDZs were evaluated in a mouse subcutaneous tumor model. In this experiment, tumors in both the normal saline group and the Fe/ZIF-8 (CZ) treatment group showed similar growth without inhibition. Treatment with DZs resulted in a 56.9% reduction in tumor volume, while a significant 90.8% reduction in tumor volume was observed after treatment with CDZ. Thus, CDZs had a marked anti-HCC effect and the efficacy of CDZs was significantly superior to that of DZS. The homogeneous aggregation of NPs induced by the cell membrane and the improvement of the stability of the NPs minimize drug loss and promote the antitumor efficacy of CDZs.

Fytory et al. (2021) constructed new types of MOFs based on Zr ion and UiO-66- NH_2_, which loaded DOX, and their surfaces were successively modified with folic acid (FA), lactonic acid (LA) and glycyrrhetinic acid (GA). Compared to the control group, treatment of HepG2 cells with DOX loaded in NMOF could stimulate cell death. The apoptosis rates of the single-ligation group (LA) and the double-ligation group (LA+GA) were 30.1% and 29%, respectively. The *in vitro* inhibitory activity experiment in HepG2 cells showed that the inhibitory ability (IC_50_) of the free DOX group, the NMOF group, the FA-NMOF group, the LA-NMOF group, the GA-NMOF group and the LA-GA-NMOF group were 1.200, 5.982, 1.887, 0.641, 0.986, and 0.520 µM, respectively. The experiment showed that the apoptosis rate of HepG2 cells induced by double ligation NMOF was higher than in the DOX treatment group. The results of this study further demonstrated the superiority of dual connectivity NMOFs as a drug delivery system for the treatment of HCC.

Liu et al. (2022) developed a multifunctional DOX loading NPs UiO-66/Bi_2_S_3_@DOX using a one-step solvothermal method, the particle could simultaneously achieve a photothermal effect and pH-triggered DOX release. The combination of transcatheter arterial chemoembolization (TACE) and PTT significantly inhibited tumor growth. Histopathological analysis showed extensive necrosis, decreased regulation of angiogenesis, and increased apoptosis in treated HCC. These results indicate that the nanosystem platform UiO-66/Bi_2_S_3_@DOX is a promising therapeutic agent to improve the TACE treatment of HCC.

The anticancer properties of UiO-66/Bi_2_S_3_@DOX were investigated in N1S1 tumor-bearing rats. MRI showed that there were no significant differences in preoperative tumor volume among seven treatment groups (total of 55 rats) in the study. On day 10 after the operation, the tumor volumes in the phosphate buffered saline solution (PBS), PBS+NIR, DOX, UiO-66/Bi_2_S_3_, UiO-66/Bi_2_S_3_@DOX, UiO-66/Bi_2_S_3_+NIR, and UiO-66/Bi_2_S_3_@DOX+NIR treatment groups were 8136 ± 799.5, 8043 ± 736.0, 4740 ± 954.9, 8461 ± 788.5, 4729 ± 658.3.0, 5219 ± 770.5, and 2826 ± 842.3 mm^3^, respectively. The tumor growth rate in the PBS group, the PBS+NIR group, and the UiO-66/Bi_2_S_3_ group on day 10 was higher than in the other four treatment groups, showing a poor inhibitory capacity of tumor volume. There were no significant differences between the DOX, UiO-66/Bi_2_S_3_@DOX, and the UiO-66/Bi_2_S_3_+NIR group, but tumor suppression in the UiO-66/Bi_2_S_3_@DOX+NIR group was significantly better than any other group. Moreover, compared to other groups, the average tumor weight in the UiO-66/Bi_2_S_3_@DOX+NIR group was the lowest, further demonstrating that the combination achieved better tumor inhibition. In conclusion, this *in vivo* antitumor study confirmed that simultaneous PTT and chemotherapy produce synergistic enhancement effects that cannot be achieved with a single treatment approach.

Samui et al. (2019) developed a NH_2_-MIL-53(Al)NMOF modified with LA and loaded with DOX. HepG2 cell line has high expression of the asialoglycoprotein receptor (ASGPR), and LA has strong binding affinity to this receptor, thus NMOF modified with LA achieves better anti-HCC activity. In their study, MTT analysis showed that NH_2_-MIL-53(Al)NMOF achieved stronger cytotoxicity against the HepG2 cell line compared to the normal cell line.

**FIGURE 5:**
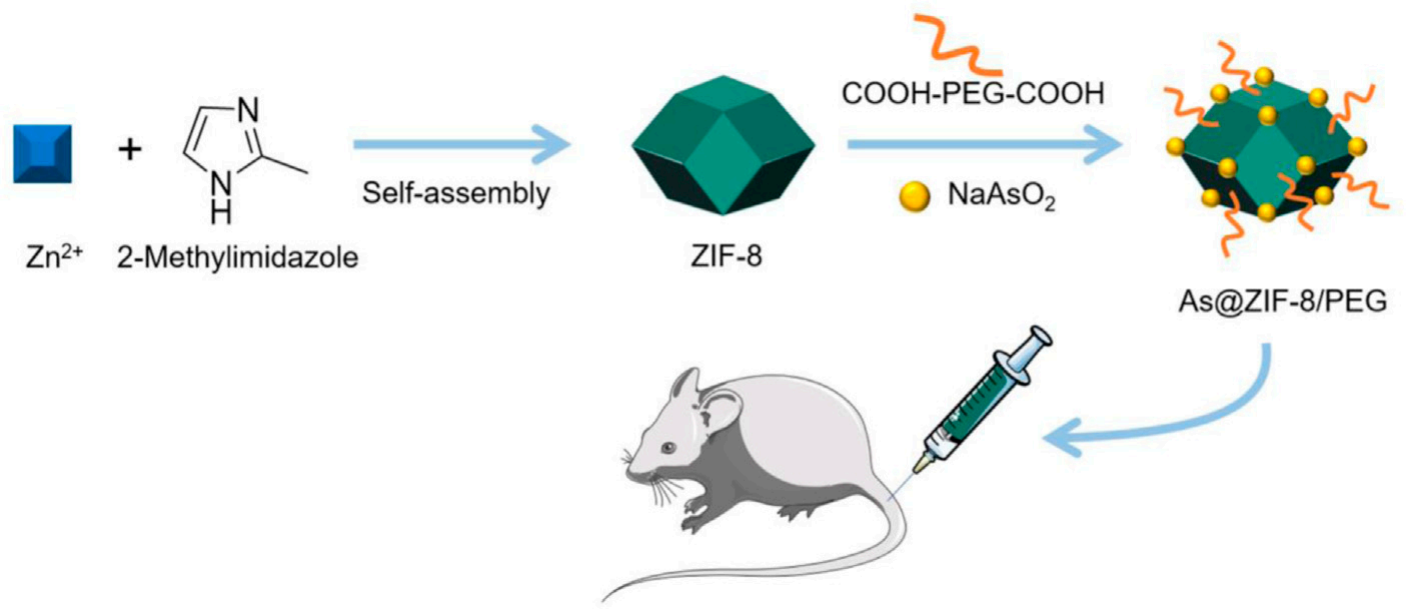
Synthesis and application of As@ZIF-8/PEG NPs. The schematic pieces were provided by Smart Medical Art and adapted [http://www.servier.com]. Servier Medical Art by Servier is licensed under a Creative Commons Attribution 3.0 Unported License.

In another study, Chen et al. (2022) used ZIF-8 as the backbone and loaded with arsenic trioxide (ATO) to develop the NPs As@ZIF-8. These NPs were then encapsulated in COOH-PEG-COOH to obtain As@ZIF-8/PEG NPs, which improved their pharmacokinetic properties (Figure 5). A mouse subcutaneous tumor model was used to test the activity of As@ZIF-8/PEG. Mice were randomly divided into four groups (five mice in each group) until tumors grew to 200–400 mm^3^. The first group was the control group with only insufficient radiofrequency ablation (IRFA) treatment. The second group was treated with IRFA and free ATO. The third group was treated with IRFA+As@ZIF-8/PEG NPs and the fourth group with IRFA+ZIF-8 NPs. The tumor growth curve shows that the inhibitory effect of the As@ZIF-8/PEG NPs-treated group on residual tumor growth was the most obvious, while that of the ZIF-8 nanocarrier was almost negligible. It should be noted that ATO also partially delayed tumor growth, but the antitumor effect of ATO was lower than that of the control group As@ZIF-8/PEG NP. The anatomical image of the tumor tissue on day 21 showed that the tumor volume in the As@ZIF-8/PEG NP group was lower than in the other groups, and the tumor volume of the free ATO group was lower than that of the control group, but much larger than that of the As@ZIF-8/PEG NP group. This experiment indicated that As@ZIF-8/PEG NPs combined with IRFA could significantly improve the therapeutic effects of IRFA.

Cheng et al. (2019) prepared magnetic nanocomposite Fe_3_O_4_-ZIF-8 carrying DOX for the treatment of HCC. The Cell Counting Kit-8 (CCK-8) assay and flow cytometry were used to determine the inhibitory effects of Fe_3_O_4_-ZIF-8, DOX and DOX@Fe_3_O_4_-ZIF-8 on MHCC97H cells. The results of the CCK-8 assay showed that Fe_3_O_4_-ZIF-8 was not toxic to MHCC97H cells, DOX@Fe_3_O_4_-ZIF-8 had an obvious inhibitory effect on MHCC97H cells. The cell uptake test showed that DOX@Fe_3_O_4_-ZIF-8 accumulated in the cytoplasm and nucleus, possibly because nanoscale DOX@Fe_3_O_4_-ZIF-8 can easily cross the cell membrane. Furthermore, due to effective drug accumulation, DOX@Fe_3_O_4_-ZIF-8 can induce apoptosis of MHCC97H cells. In sum, compared to free DOX, DOX@Fe_3_O_4_-ZIF-8 had a stronger effect on HCC cells, indicating that it has the potential to become a chemotherapeutic drug for HCC.

Li Z et al. (2022) developed a triptolide (TPL)-loaded MOF (TPL@CD-MOF) based on the cyclodextrin (CD) structure, which improves the solubility and bioavailability of TPL, thus enhancing its inhibitory effect on HCC. In the Huh-7 subcutaneous xenograft tumor model, the antitumor activity of TPL@CD-MOF was investigated. The results showed that compared to the normal saline group or the free TPL group, the TPL@CD-MOF group produced better antitumor efficacy and the tumor volume and tumor weight of this group were lower.

Bieniek et al. (2021) prepared a pocket wheel framework (PPF) based on Zn (NO_3_)_2_•6H_2_O and TCPP and loaded it with sorafenib (SOR) to obtain SOR@PPF. In different proportions of ethanol aqueous solutions, SOR was deposited on PPF in two different sizes with different resolution rates, i.e., slow released (SR) and fast released (FA). In the *in vitro* anti-HCC cell activity assay, the concentration for 50% of maximal effect (EC_50_) of SOR alone was 13.8 μM and 10.0 mΜ after 24- and 72-h administration, respectively. In contrast, the EC_50_ of SOR@PPF was 1.6 µΜ and 1.81 µΜ after 24 and 72 h of administration, respectively. In *in vivo* experiments, compared to the control group, both SR-SOR@PPF and FR-SOR@PPF effectively inhibited distant metastasis and *in situ* cancer recurrence after operation. Compared to the control group, in rats treated with SR-SOR@PPF, the odds ratios (OR) of distant metastasis and *in situ* recurrence were 0.26 and 0.38, respectively. In the rats treated with FR-SOR@PPF, the OR values of distant metastasis and *in situ* recurrence were 0.56 and 0.63, respectively. These results indicated that the antitumor effect of SOR@PPF improved significantly compared to free SOR. In addition, by controlling PPF degradation and sorption, the antitumor effect of SOR *in vitro* and *in vivo* can be regulated.

**FIGURE 6:**
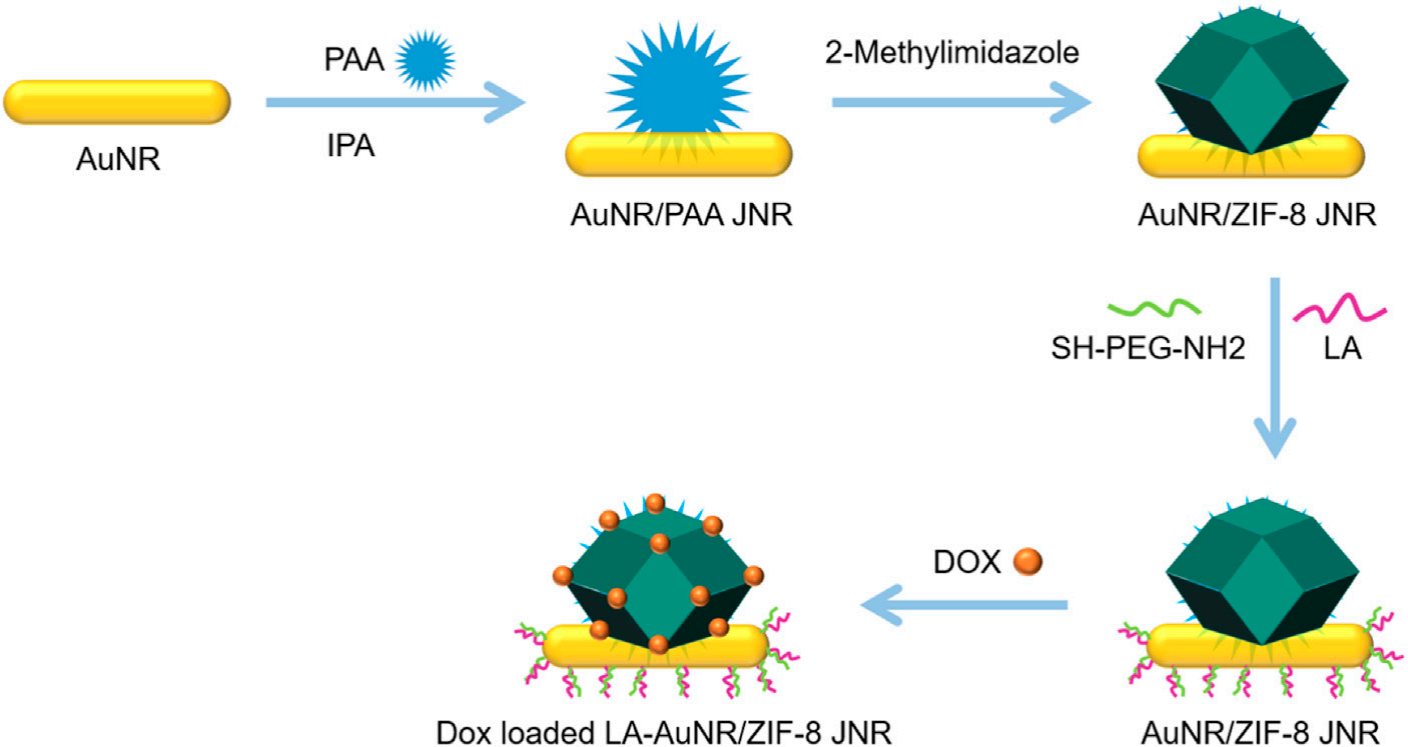
Synthesis of NPsLA-AuNR/ZIF-8 JNPs.

Zhang et al. (2021) mixed one-dimensional Au nanorod (AuNR) with PAA, aqueous ammonium hydroxide solution (NH_3_•H_2_O), and isopropanol (IPA) to obtain AuNR/PAA JNPs. Zinc nitrate and imidazole (Hmim) were then added to form ZIF-8 on the PAA side of AuNR/PAA JNPs and AuNR/ZIF-8 JNPs were obtained. Finally, the exposed side surface of AuNR was modified with LA to obtain the LA-AuNR/ZIF-8 JNPs targeting HCC (Figure 6). These NPs could load about 30 wt% DOX and achieved better and faster drug release under the NIR laser and pH 5.3. *In vivo* studies using mice xenografted with H-22 tumor cells showed that the JNPs+laser group (808 nm) caused more cancer cell death than the pure JNPs group, which indicated that JNP could act as a photothermal agent to effectively kill cancer cells under NIR laser irradiation. The tumor inhibition rate of the JNP+DOX+laser group was as high as 93%, which was higher than that of the JNP+DOX and JNP+laser groups. These findings indicated that LA-AuNR/ZIF-8 JNPs, combined with drugs and lasers, could cause synergistic chemotherapy and PTT effects, thus achieving better therapeutic effects.

Liu et al. (2021b) loaded the ferroptosis inducer SOR, which is used to treat advanced HCC, onto the Fe metal organic framework [MIL-101 (FE)] to prepare MIL-101(Fe)@SOR NP. After 60 h of administration, the drug release of these NPs reached approximately 35% at pH 5.5 and only 10% at pH 7.4. These NPs significantly induced ferroptosis in HepG2 cells and decreased the concentration of glutathione and glutathione peroxidase 4 (GPx-4). The results of *in vivo* experiments showed that MIL-101(Fe)@SOR NPs could significantly inhibit tumor progression, reduce the expression level of GPx-4, and the long-term toxicity was negligible. Subsequently, to enhance the nanodrug tumor targeting and penetration capabilities, an iRGD peptide (amino acid sequence: CRGDK/RGPD/EC) was introduced containing a tumor-homing motif (RGD) and a tissue penetration motif (CendR).

*In vivo,* mice with implanted H-22 tumor cells exhibited extremely rapid tumor growth in the control group and MIL-101(Fe) NPs group, while in other groups, the growth was slow. In the MIL-101(Fe)@SOR+iRGD group, the tumor was the smallest. Compared to other groups, mice treated with MIL-101(Fe)@SOR+iRGD had the highest tumor inhibition and significantly reduced tumor weight. Additionally, during the treatment period, the weight of the mice in the MIL-101(Fe)@SOR+iRGD group did not decrease significantly. Tumor sections were stained and imaged to compare with other groups, the necrosis area of tumor tissue in the MIL-101 (Fe)@SOR+iRGD group was the largest, and the number of GPX-4 positive cells was the lowest. These experimental results show that MIL-101(Fe)@SOR+iRGD has relatively optimal tumor inhibition.

**FIGURE 7:**
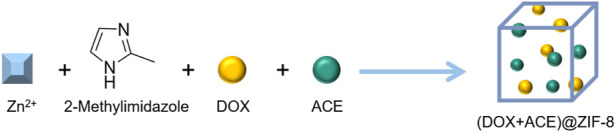
Synthesis of (DOX+ACE)@ZIF-8.

Jing et al. (2021) prepared a nanocarrier zeolite imidazoline framework (ZIF-8) by the one-pot method and loaded DOX and acetazolamide (ACE) simultaneously to obtain (DOX+ ACE)@ZIF-8. The DOX and ACE drug loading efficiencies were 7.29% and 4.62%, respectively (Figure 7). *In vitro* cell inhibitory activity experiments showed that the inhibitory rate (IC_50_) of blank ZIF-8 in the human normal liver cell line HL7702 was greater than 100 μg/ml, which indicates that the cytotoxicity of ZIF-8 is low. The antitumor effect of (DOX+ACE)@ZIF-8 was dose dependent and the inhibitory capacity (IC_50_) against Walker 256 cells was 2.36 μG/ml and 0.66 μG/ml (corresponding to ace and DOX), respectively. The safety of (DOX+ACE)@ZIF-8 *in vivo* was experimentally evaluated. The hemolytic potential of (DOX+ACE)@ZIF-8 in 50–200 μG/ml is negligible (<5%). Rats injected with (DOX+ACE)@ZIF-8 by a single intratumoral dose or intravenous injection showed little damage to normal tissues or adverse hematological effects, which preliminarily demonstrated the high biocompatibility of (DOX+ACE)@ZIF-8.

## Application of MOF in tumor imaging

Some MOFs have specific photosensitive properties and can shine under excitation at a specific wavelength, enabling MOFs to possess the ability of tumor imaging, which is a very important property in identifying and treating tumors (He et al., 2019; Huang et al., 2021).

Sun et al. (Sun Q.X et al., 2021) prepared MOF-RB by loading rhodamine B (RB) into a common MOF structural unit UiO-66-NH_2_ (Zr-MOF). MOF-RB was used to perform confocal laser scanning microscopy (CLSM) fluorescence imaging and inductively coupled plasma mass spectrometry (LA-ICPMS) laser ablation under elemental imaging on the same group of HepG2 cells in the designated area. This dual-mode imaging strategy helps visualize the migration of copper transporter 1 (CTR1), while providing clear information on the migration and redistribution of CTR1 during exposure to divalent copper/cisplatin through joint imaging of CLSM and LA-ICPMS on the same group of HepG2 cells. This dual-mode imaging strategy provides extremely valuable information to elucidate biological processes related to CTR1.

**FIGURE 8:**
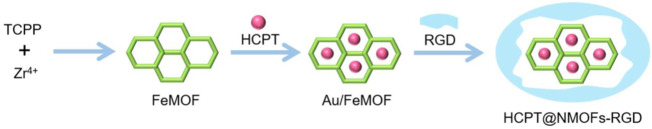
Synthesis of HCPT@NMOFs-RGD.

Shang et al. (2022) prepared a zirconium porphyrin metal organic framework (NMOFs) and then loaded 10-hydroxycamptothecin (HCPT) into the pores of the NMOFs and wrapped it with arginine glycine aspartic acid (RGD) peptide to obtain a new nanocomposite HCPT@NMOFs-RGD (Figure 8). In a mouse tumor model with xenograft, the antitumor activity of HCPT@NMOFs-RGD was evaluated. NMOFs-RGDs had low toxicity, good biocompatibility, and strong imaging ability. In a zebrafish HCC model, specific binding of HCPT@NMOFs-RGD with the integrin α_v_β_3_ and enrichment in tumors lead to a reduction in tumor volume. In the mouse xenograft tumor model, after 12 days of HCPT@NMOFs-RGD treatment, the tumor size of the NMOFs group (PDT treatment) and the HCPT group (chemotherapy) was smaller than that of the blank control group. Importantly, tumors in the HCPT@NMOFs-RGD group were significantly reduced and even disappeared in one sample. Tumor weights in the NMOF group, the HCPT group, and HCPT@NMOFs-RGD all decreased significantly. These data prove the advantage of synergistic antitumor of HCPT@NMOFs-RGD.

Chen et al. (2019) synthesized a new type of NPs, namely, folic acid nanoscale gadolinium porphyrin metal organic frameworks (FA-NPMOF), based on a gadolinium porphyrin-based MOF and then combined with folic acid (FA). Subsequently, the biological toxicity and imaging ability of FA-NPMOF were measured using HepG2 cells, zebrafish embryos and larvae. *In vitro* cell experiments, HepG2 cells were found to have significant apoptosis after treatment with PDT (655 nm) with FA-NPMOF. In *in vivo* experiments in zebrafish, HCC cells were necrotic and triggered inflammatory reactions. However, enhanced green fluorescent protein (EGFP) fluorescence, thermal imaging, and tumor shrinkage also verified its therapeutic effect. These experiments showed that FA-NPMOF achieved a good therapeutic effect on HCC *in vitro* and *in vivo*. The key information of the MOFs mentioned above is listed in Table 1.

**TABLE 1:** Key characteristics of the MOFs involved in this article.

MOFs	Metal	Component	Drug	Surface functionalization	Ref
MINPs	Mn^2+^	porphyrin, ICG	—	—	Shi et al. (2018)
ZIF-8@Ce6–HA	Zn^2+^	2-Methylimidazole	Ce6	HA	Fu et al. (2020)
DOX@NPMOF	Zr^2+^	TCPP	DOX	—	Liu et al. (2017)
PEG-Au/FeMOF@CPT	Zr^2+^	TCPP	CPT	Au NPs,C_12_SH,PEG-SH	Ding et al. (2020)
CDZs	Fe^2+^, Zn^2+^	2-Methylimidazole	DHA	Cancer cell membrane	Xiao et al. (2020)
LA-GA-NMOF	Zr^4+^	UiO-66-NH_2_	DOX	FA, LA, GA	Fytory et al. (2021)
UiO-66/Bi_2_S_3_@DOX	Bi^3+^	UiO-66	DOX	—	Liu et al. (2022)
NH2-MIL-53(Al)NMOF	Al^3+^	2-Aminoterephthalic acid	DOX	LA	Samui et al. (2019)
As@ZIF-8/PEG	Zn^2+^	2-Methylimidazole	ATO	COOH-PEG-COOH	Chen et al. (2022)
DOX@Fe_3_O_4_-ZIF-8	Fe^2+^, Zn^2+^	2-Methylimidazole	DOX	—	Cheng et al. (2019)
TPL@CD-MOF	K^+^	CD	TPL	—	Li Z et al. (2022)
SOR@PPF	Zn^2+^	TCPP	SOR	—	Bieniek et al. (2021)
LA-AuNR/ZIF-8	Zn^2+^	2-Methylimidazole	DOX	Au nanorod, LA	Zhang H et al. (2019)
MIL-101(Fe)@SOR	Fe^3+^	2-Aminoterephthalic acid	SOR	—	Liu et al. (2021b)
(DOX+ACE)@ZIF-8	Zn^2+^	2-Methylimidazole	DOX, ACE	—	Jing et al. (2021)
MOF-RB	Zr^2+^	UiO-66-NH_2_	RB	—	Sun Q.X et al. (2021)
HCPT@NMOFs-RGD	Zr^4+^	TCPP	HCPT	RGD peptide	Shang et al. (2022)
FA-NPMOF	Gd^3+^	TCPP	FA	—	Chen et al. (2019)

## Challenges of MOF in cancer treatment

Although theoretically a variety of metal ions can be used in MOF assembly, in fact—after excluding toxic metal ions—only a few ions are suitable; these include Zr (Wang et al., 2021), Fe (Wang et al., 2019; Yao et al., 2022), Zn (Farhadi et al., 2021; Wan et al., 2021), Mn (Lan et al., 2018; Li et al., 2018), and Cu (Lan et al., 2018; Li et al., 2018), which limits the diversification of MOFs types. In addition to the limitation of the metal ion type, the cytotoxicity caused by the physicochemical properties of the MOFs is also an important factor limiting the application of MOFs (Xia et al., 2021; Xie et al., 2021). These key physicochemical properties, including size distribution, shape, and surface hydrophobicity, affect the solubility of MOFs. To overcome these problems, a large number of studies have been carried out in recent years to modify the surface of MOFs with hydrophilic groups or prepare MOFs as liposomes (Zhang D et al., 2019; Bao et al., 2020; Wang et al., 2021). These works are of great practical value.

Another problem that restricts the use of MOFs in anti-HCC treatment is drug loading and drug release (Gharehdaghi et al., 2021; Jiang et al., 2021). The drug loading and release properties of these nanodrug delivery platforms depend on both the structure of the MOFs and the properties of the loaded drugs. It is challenging to overcome this dilemma. The structure of MOFs needs to be chemically modified according to the different types of loaded drug to match the carrier and drug and achieve a more suitable dissolution. MOFs have a large molecular weight, and *in vivo* metabolic limitations resulting from mass drug administration are also a problem worthy of follow-up research (Lakshmi and Kim, 2019; Chen J et al., 2021).

## Conclusion

The application of MOFs in breast cancer (Qin et al., 2020; Xu et al., 2020; Alves et al., 2021; Zhou et al., 2021) and cervical cancer (Sava Gallis et al., 2017; Zhao et al., 2018; Rao et al., 2022) is relatively mature, while in HCC it is still in its infancy. This is mainly because the pathogenesis of HCC is complex and there are less drugs available for HCC treatment. In this paper, we reviewed the latest progress of MOFs in HCC therapy, including the latest research progress in direct use of MOFs as anti-HCC treatment and in combination with other drugs. As a porous material, the MOF not only has photodynamic and photothermal properties but also has the advantages of high porosity, adjustable structure, versatility, and biocompatibility. Through synergistic treatment of photodynamic and photothermal effects, MOFs has shown outstanding effects in various solid tumor treatment. On the basis of the macroporous structure of the MOFs, chemotherapy drugs such as DOX, CPT, and SOR have been used to construct drug-loaded NPs with MOFs to cooperate with photodynamic, photothermal, chemical dynamics, and other methods to treat HCC. According to the reported cases, ZIF-8, UiO-66 and porphyrin MOFs are the most common materials. Porphyrin MOFs possess PDT and PTT effects, while MOFs such as ZIF -8 and UiO-66 mainly be used as the drug delivery platform to HCC treatment.

In HCC chemotherapy, the most problem is drug resistance and toxicity caused by excessive administration. MOFs can be administered through TACE technology to create new possibilities of local drug delivery and controlled release, while avoiding the toxicity of chemotherapeutics drugs. In addition, the new therapeutic mechanisms of PDT, PTT and CDT can resolve the problem of drug resistance. It is hoped that some stimuli-responsive (pH, sound and thermal) MOFs will be applied to HCC treatment in the future, so as to diversified treatment strategies. Most of the reported MOFs are constructed with known ligand structures because the application of MOFs is still in the exploratory stage. With further research, more advanced structures will be used to construct different MOFs. It is expected that these new developments will bring a new situation to HCC treatment.

## Data Availability

The original contributions presented in the study are included in the article/supplementary material, further inquiries can be directed to the corresponding author.
